# Adhesion and whitening effects of P11-4 self-assembling peptide and HAP suspension on bovine enamel

**DOI:** 10.1007/s00784-020-03654-1

**Published:** 2020-10-27

**Authors:** Niloofar Hojabri, Dalia Kaisarly, Karl-Heinz Kunzelmann

**Affiliations:** 1 Department of Conservative Dentistry and Periodontology, University Hospital, Ludwig-Maximilians-University Munich, Goethestr. 70, 80336 Munich, Germany; 2 Biomaterials Department, Faculty of Oral and Dental Medicine, Cairo University, Cairo, Egypt

**Keywords:** Self-assembling peptide P11-4, Hydroxyapatite (HAP), Bovine enamel, Color measurement, Whitening effects, SEM

## Abstract

**Objectives:**

This study evaluated the adhesion and whitening effects of a combination of P11-4 self-assembling peptide and hydroxyapatite (peptide-HAP) on bovine enamel.

**Methods:**

Forty-six caries-free bovine teeth were selected, and 40 teeth were randomly allocated to one of five groups (*n* = 8). First, the effects of application frequency, exposure time, and storage in saliva on the whitening effects of an experimental low-concentrated peptide-HAP suspension (0.5 wt% HAP; Curodont, Credentis) were evaluated and compared with a commercial bleaching agent (VivaStyle Paint on Plus, VS, Ivoclar Vivadent). Tooth color was measured using a spectrophotometer (Gretag MacBeth), and color changes ΔE were statistically analyzed. Second, the effects of peptide-HAP concentration (low versus high: 6.25% HAP; Curodont Protect), and its interactions with saliva and postapplication restaining, were investigated. Third, enamel surfaces (*n* = 2) were treated with low concentration peptide-HAP and high-concentration peptide-HAP in polymeric and monomeric forms (Curodont Protect & Curodont Repair, Credentis) and analyzed by SEM.

**Results:**

The Δ*E* of the low-concentration peptide-HAP suspension did not differ from that of VS. Application frequency, exposure time, and storage in saliva did not have any significant impact on whitening efficacy of the peptide-HAP suspension. Increasing the concentration of the suspension did not promote overall Δ*E*. SEM observations confirmed the presence of the newly generated peptide and HAP on the enamel surface.

**Conclusions:**

The peptide-HAP suspension is a mild tooth whitener, and the adhesion of peptide-HAP to enamel is concentration dependent.

**Clinical relevance:**

This peptide-HAP suspension is effective in offsetting discoloration caused by restaining after treatment.

## Introduction

A number of social and psychological studies have shown that physical appearance notably influences quality of life [1]. In particular, tooth discoloration is a widespread esthetic concern for many individuals [2]. As a consequence, tooth whitening and attaining an ideal esthetic result is becoming an increasingly important issue in modern cosmetic dental practice. Most tooth whitening products are based primarily on hydrogen peroxide (H_2_O_2_) or one of its precursors, carbamide peroxide, which influences tooth color by chemical reactions [3]. However, several clinical studies have shown that using such whitening agents may cause undesirable side effects, including cervical root resorption and tooth sensitivity [4]. As a result, other tooth whitening alternatives are being developed.

One of the recently proposed whitening agents is hydroxyapatite (HAP), which is the main component of dental hard tissues and bone and can be artificially synthesized [5]. HAP is used in dental treatments, such as for preventing enamel caries, restoring initial cavities, nano-hydroxyapatite-containing glass ionomer cements, and removing dental plaque in addition to hydroxyapatite-containing tooth pastes [6–9].

HAP may contribute to tooth whitening because it is a white biomaterial that adheres to the tooth surface, and the newly generated HAP layer leads to diffuse reflection, causing the tooth to appear brighter [10]. After treatment of demineralized enamel, a new homogenous thick HAP layer covering the tooth surface contributes to increasing the diffuse reflection of the light and leads to a measurable enhancement of lightness/brightness [11]. However, the whitening efficacy of such agents depends on the level of adhesion of the HAP particles.

Peptide self-assembly is a relatively new approach for building synthetic super molecular architectures [12], which refers to the organization of peptides with other peptides having a similar structure in multimeric assemblies by spontaneous and reversible noncovalent interactions [13, 14]. The P11-4 peptide with the sequence Gln-Gln-Arg-Phe-Glu-Trp-Glu-Phe-Glu-Gln-Gln responds to pH triggers and assembles in the hierarchical order of tapes, ribbons, fibrils, and fibers. In particular, for a pH lower than 7.5, P11-4 forms ß-sheet, tape-like assemblies [15]. When the pH decreases, the peptide transfers from a fluid phase to a nematic gel.

P11-4 can be applied as templates or scaffolds in tooth remineralization. The scaffolds support in situ nucleation of calcium phosphate [13]. A negatively charged residue that is acidic, such as Glu, can serve as nucleation sites for calcium ions, while an acidic residue that is positively charged, such as Arg, may be able to interact with phosphate ions. If the side chains of glutamic acid and arginine interact with dissolved calcium and phosphate to enhance the nucleation of calcium phosphate combinations, they may also interact with the enamel surface [16–18].

Teeth are remineralized primarily by the delivery of phosphate and calcium ions into tooth cavities. Phosphate and calcium ions may be present in different crystalline forms (e.g., HAP-based materials) or as amorphous calcium phosphate (e.g., Recaldent-based materials) [13]. Recently, an alternative approach to tooth remineralization was introduced that is based on rationally designed self-assembling peptides [14, 19].

We hypothesized that interaction between the calcium phosphate particles (HAP) mixed with dissolved P11-4 self-assembling peptide and the tooth surface will enhance the adhesion of the HAP particles to the tooth surface, improving the whitening efficacy by causing diffuse reflection from the newly generated HAP layer.

The aim of this study was to evaluate the effects of exposure time, application frequency, and storage in saliva on the degree of color change caused by an aqueous suspension of 0.02 mg/ml P11-4 and 0.5 wt% HAP compared with an existing commercial peroxide-based bleaching agent. Furthermore, low and high concentrations of peptide-HAP suspension were compared, and the impact of storage in saliva and restaining on color changes caused by the suspensions were evaluated. Structural changes in bovine enamel surface after treatment with the peptide-HAP suspension were qualitatively analyzed by SEM. The null hypothesis states that there is no difference regarding application frequency, exposure time, or concentration of peptide-HAP suspension on the color changes of enamel. Furthermore, the null hypothesis states that there is no difference between the peptide-HAP solution and a commercial bleaching agent regarding color changes of enamel.

## Materials and methods

Forty-six freshly extracted bovine incisors without roots, any stains, cracks, or caries were randomly selected and polished using a rubber cup in a dental hand piece for 30 s with a fine prophylactic polishing paste (Proxyt, RDA 7, fine, Ivoclar Vivadent, Schaan, Liechtenstein). Forty samples were assigned to the color measurement experiments, and six samples were assigned to SEM analysis.

### Sample preparation for color measurement

Teeth were embedded in resin (Technovit 4004 transparent embedding kits, Kulzer, Germany) using silicon molds (2.4 cm × 2 cm × 1.5 cm). The superficial enamel of the samples was ground to obtain a flat surface. For standardizing the color measurement area, the vestibular surface of each sample was polished in a way that an ellipse of enamel with a vertical axis of approximately 10 mm and a horizontal axis of approximately 6 mm was exposed. The samples were polished under water cooling with 600- and 1200-grit SiC abrasive papers (Leco Corporation, St. Joseph, USA) which correspond to polishing particles of an average size of 16 μm and 6.5 μm, respectively. The samples were stored in standard mineral water (Evian; Danone Waters Deutschland, Frankfurt, Germany) at room temperature until the experimental procedures.

Subsequently, samples were immersed into a staining solution for 72 h at room temperature. The staining solution was a mixture of 5 g each of black tea (Teekanne GmbH, Düsseldorf, Germany), dark soy sauce (Premium dark soy sauce, a Chinese brand), espresso (Nescafe type espresso, Nestle AG, Frankfurt am Main, Germany), and Maggi sauce (Maggi Würze, Maggi GmbH, Germany). Each component was added to 100 ml of water at 95 °C and left to cool for 20 min before use, at which point all solutions were mixed together [20]. The staining solution was filtered with a cellulose filter made of natural fibers (Teefilter, size 3, Profissimo, dm-drogerie markt, Karlsruhe, Germany). The pH of the staining solution was set at 5.5 at 23 °C using an electronic pH meter (WTW bench pH/mV meters Routine meter pH 526, Sigma-Aldrich Chemie GmbH, Taufkirchen, Germany). After removing the teeth from the staining solution the samples were rinsed with water and blot dried with a paper towel to remove loose extrinsic colorants followed by color measurement to obtain the baseline values.

### Treatment procedures

Prepared samples were randomly assigned to one of five groups (*n* = 8) according to the treatment solutions, which consisted of different concentrations self-assembling peptide P11-4 and HAP (peptide-HAP). Experimental groups differed with respect to exposure time, application frequency, and restaining.

The first and third groups were treated with an experimental aqueous suspension of 0.02 mg/ml P11-4 peptide and 0.5 wt% HAP (Curodont, Credentis, AG, Windisch, Switzerland), and the second group was treated with a commercial bleaching agent (VivaStyle Paint On Plus, Ivoclar Vivadent, Schaan, Liechtenstein) consisting of 6% hydrogen peroxide solution. The fourth group was treated with a high concentration peptide-HAP suspension of 5 ml P11-4 and 6.25 wt% HAP (Curodont Protect, Credentis AG, Windisch, Switzerland). The fifth group served as the control group in which only water was applied.

### Effect of exposure time and application frequency

First, we investigated the effect of application frequency, exposure time, and storage in saliva on the degree of color change caused by the suspension compared with a bleaching agent. In the first group, gp-1-PH/lc, the baseline color (T0/0) was measured and a low-concentration aqueous peptide-HAP (PH/lc) suspension was applied once for 30 s using a microbrush. After 1 min of exposure time (T1min/1×), the sample was rinsed with water for five seconds and blot dried with a soft absorbent paper towel (half dry) before the color was measured. After 5 min of exposure (T5min/1×), the third color measurement was taken. Subsequently, the solution was applied four more times, and after each application, we rinsed the sample with water and blot dried it. Sample color was measured 1 (T1min/5×) and 5 (T5min/5×) min from the last application. Thereafter, samples were immersed in artificial saliva and stored in an incubator at 37 °C for 24 h, followed by color measurement (T24h). Table 1 summarizes the treatment conditions and steps of the samples across all groups. The artificial saliva was composed of potassium chloride (1.2 g), sodium chloride (0.84 g), di-potassium hydrogen phosphate (0.26 g), calcium chloride (0.14 g), and water (1000 g) (Pharmacy of the LMU Munich, Munich, Germany).

**Table 1:** Treatment steps and conditions in all groups

Treatment group	Exposure time and application frequency	Treatment agent				Number of received treatments		Exposure time			Storage in saliva	Restaining
		Aqueous suspension P11-4 and HAP^*^	VivaStyle Paint On Plus	Curodont Protect and 6.25% HAP**	Water	1×	5×	1 min	5 min	10 min	24 h	72 h
gp-1-PH/lc	T1min/1×	✓				✓		✓				
	T5min/1×	✓				✓			✓			
	T1min/5×	✓					✓	✓				
	T5min/5×	✓					✓		✓			
	T24h/5×	✓					✓		✓		✓	
gp-2-VS	VS10min		✓							✓		
	VS24h		✓							✓	✓	
gp-3-PH/lc		✓					✓				✓	✓
gp-4-PH/hc				✓			✓				✓	✓
gp-5-WA					✓		✓				✓	✓

*Low-concentrated aqueous suspension (0.02 mg/ml P11-4 and 0.5 wt% HAP)

**High-concentrated protein suspension (Curodont Protect and 6.25% HAP)

In the second group, gp-2-VS, a thin layer of the bleaching agent VivaStyle Paint On Plus (VS) was directly applied to the samples using a microbrush and was left for 10 min to allow undisturbed interactions with the surface following the manufacturer’s instructions. Then, the dried varnish was removed using a scalpel and a soft toothbrush without any tooth paste. The sample was rinsed thoroughly with water and blot dried with a soft paper towel, and its color was measured (VS10min). The sample was immersed in artificial saliva for 24 h followed by another color measurement (VS24h).

### Effect of peptide-HAP concentration

The second part of the experiment compared the impact of two different concentrations of peptide-HAP-containing suspensions, storage in saliva, and restaining for 72 h on the whitening efficacy. The third group, gp-3-PH/lc, was treated with the experimental low-concentration peptide-HAP suspension, as in the first group. The fourth group, gp-4-PH/hc, was treated with a high-concentration peptide-HAP suspension (PH/hc), and the fifth group, gp-5-WA, served as the control group in which only water (WA) was applied (Table 1).

After measuring the baseline colors, the corresponding treatment suspensions (water in group-5) were applied to sample surfaces for 30 s with a microbrush. After 5 min of exposure time, the samples were rinsed with water and blot dried with a paper towel (half dry), and the color was measured. Subsequently, samples were stored in artificial saliva at 37 °C in an incubator for 24 h followed by repeated color measurement and immersion in the staining solution for an additional 72 h, followed by rinsing with water, blot drying, and another color measurement.

### Color measurement

Sample color was determined using the Color Eye 7000 spectrophotometer (Gretag MacBeth, X-Rite, Munich, Germany). To eliminate associated errors, this experiment was performed in the absence of light. Thus, the room was darkened during measurements per the spectrophotometer’s recommendations.

Sample color was evaluated according to the CIE L*a*b* color scale; in this system, L* represents lightness/brightness, a* defines the red-green value, and b* denotes the yellow-blue value. Differences between L*, a*, and b* between baseline readings and after each experimental step were expressed as ∆L*, ∆a*, and ∆b*. Accordingly, overall color difference (*∆E*) was calculated using the following equation: $\Delta  E=\sqrt{{\left(\Delta  {L}^{\ast}\right)}^2+{\left(\Delta  {a}^{\ast}\right)}^2+{\left(\Delta  {b}^{\ast}\right)}^2}$.

### Statistical analysis

The means and standard deviations of color change were calculated and statistically analyzed using IBM SPSS statistics (version 18.0). Data were tested for normality using the Shapiro Wilk test; the level of significance for all tests was set at 0.05.

To determine whether exposure time, application frequency, or storage time in saliva had any significant impact on the overall degree of color change (*∆E*), paired *t* tests were employed. Regarding gp-1-PH/lc and gp-2-VS, comparison of the means of ∆*E* at T5min/5× and VS10min were analyzed using the Mann-Whitney *U* test, and T24h versus VS24h were analyzed using an independent *t* test.

Regarding gp-3-PH/lc, gp-4-PH/hc, and gp-5-WA, the Mann-Whitney *U* test was used to assess differences between the overall color changes. A paired *t* test was applied to analyze the effect of artificial saliva on the color change of the samples in the groups and to determine the effect of restaining on the overall color change. To evaluate the overall color change in the groups, the results were analyzed using ANOVA with post-hoc Bonferroni test.

### SEM evaluation

Six bovine incisors were randomly selected, cleaned, and stained for 72 h at 25 °C. The labial enamel surface was polished using a 1200-grit abrasive paper under water cooling. Subsequently, small blocks (5 mm × 5 mm × 1 mm) were prepared from the labial surface from comparable regions (middle one-third) of each tooth [21]. Enamel blocks were mounted onto glass slides for easier handling, and samples were assigned to three groups (*n* = 2).

All samples were cleaned with 3% NaOCl for 3 min and then rinsed with water. The first group, SEM1-PH/lc, was treated with an aqueous suspension of 0.02 mg/ml peptide and 0.5 wt% HAP (PH/lc). The second group, SEM2-PH/hcp, was treated with a mixture of self-assembling peptide in polymeric form and HAP suspension (6.25 wt%) (PH/hcp) (Curodont Protect), and it has a gel-like presentation. The third group, SEM3-PH/hcm, was treated with a mixture of self-assembling peptide in monomeric form and HAP suspension (6.25 wt%) (PH/hcm) (Curodont Repair, Credentis AG, Windisch, Switzerland), and it has a watery consistency. To initiate the polymerization of the peptide in Curodont Repair, lactic acid was added to the mixture using an Eppendorf pipette and the pH at 24 °C was optimized to be slightly acidic.

For all samples, suspensions were applied for 30 s, and samples were left to interact with the mixture for 5 min. After 24 h, samples were sputter coated with an ultrathin layer of gold-palladium alloy (SC7620, Polaron, Quorum Technologies, Kent, UK), and images of the enamel samples were taken at two magnifications (× 5000 and × 10,000) using a scanning electron microscope (SEM, ZEISS Supra 55vp, Zeiss, Oberkochen, Germany).

## Results

### Effect of exposure time and application frequency

The ΔL*, Δa*, Δb*, and Δ*E* means, standard deviations, and associated color measurements at all the treatment steps for gp-1-PH/lc are summarized in Table 2. The absolute ΔL*, Δa*, and Δb* mean values in T1min/1× were the lowest. In contrast, T5min/5× demonstrated the highest Δa* and Δb* mean values. In relative terms, T24h/5× presented the highest lightness/brightness effect, but the ΔL* mean value after this step was only slightly higher than T5min/5×.

**Table 2:** Means and standard deviations of ΔL*, Δa*, Δb*, Δ*E*, and the associated color perception for all conditions in the first group gp-1-PH/lc relative to baseline values

CIELAB	T1min/1×	T5min/1×	T1min/5×	T5min/5×	T24h/5×
ΔL* Color perception	2.86 Lighter	3.98 Lighter	3.31 Lighter	4.00 Lighter	4.40 Lighter
Δa* Color perception	− 0.75 Less red	− 1.01 Less red	− 0.94 Less red	− 1.29 Less red	− 1.23 Less red
Δb* Color perception	− 1.42 Less yellow	− 1.84 Less yellow	− 1.76 Less yellow	− 2.57 Less yellow	− 2.25 Less yellow
Δ*E*	4.12 (3.98)^a^	5.22 (5.35)^a^	4.72 (4.84)^a^	5.81 (5.42)^a^	6.42 (6.31)^a^

Neither exposure time (*p* = 0.102) nor application frequency between T1min/1× and T1min/5× (*p* = 0.199) resulted in any significant differences in color change Δ*E*. Storage in saliva for 24 h did not exert a significant influence on the color change (*p* = 0.414) when comparing Δ*E* values.

**Fig. 1:**
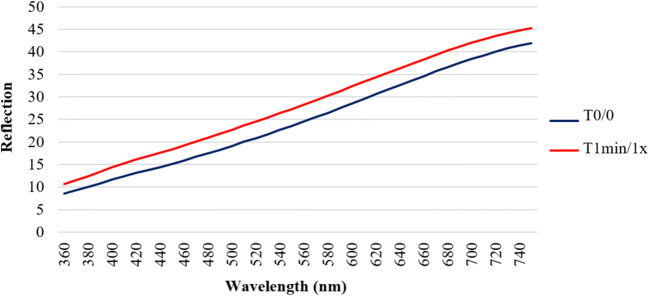
Mean reflection spectra of group-1 at T0/0 and T1min/1× over wavelengths from 360 to 750 nm

Figure 1 compares the mean reflections over the wavelengths from 360 to 750 nm for T0/0 and T1min/1×. For all given wavelengths, T1min/1× presented higher reflections than T0/0. In relative terms, a single application of the suspension after 1 min of exposure time resulted in a stronger reflection compared with baseline values.

To assess the competitiveness of the new formulation, we compared color changes caused by the low-concentration peptide-HAP suspension with the commercial home bleaching agent VivaStyle. The ΔL*, Δa*, Δb*, and Δ*E* means; standard deviations; and associated color measurements of gp-2-VS are listed in Table 3. Both suspensions demonstrated perceptible color differences, especially ΔL*, in terms of lightness/brightness, which revealed greater values in gp-2-VS than in gp-1-PH/lc. The mean ΔL* value in gp-1-PH/lc at T5min/5× (4.00) was lower than that in VS10min (5.73). After storage in saliva for 24 h, the mean ΔL* value of gp-2-VS (6.73) was higher than the mean ΔL* value obtained from gp-1-PH/lc (4.40).

**Table 3:** Mean values of ΔL*, Δa*, Δb*, and the associated color perception for the first and second groups, gp-1-PH/lc and gp-2-VS, after application and storage for 24 h

CIELAB	gp-1-PH/lc-T5min/5×	gp-2-VS10min	gp-1-PH/lc-T24h/5×	Gp-2-VS24h
ΔL* Color perception	4.00 Lighter	5.73 Lighter	4.40 Lighter	6.73 Lighter
Δa* Color perception	− 1.29 Less yellow	− 0.99 Less yellow	− 1.23 Less yellow	− 2.51 Less yellow
Δb* Color perception	− 2.57 Less red	− 1.36 Less red	− 2.25 Less red	− 3.99 Less red
Δ*E*	5.81 (5.42)a	6.72 (8.46)a	6.42 (6.31)a	9.01 (8.07)a

Matching letters for pairs of columns (first two columns and last two columns) denote no statistically significant difference between them

The overall color changes, ∆*E*, caused by the agents before and after storage in saliva for 24 h were calculated relative to their corresponding baseline values. In both conditions, for gp-2-VS, VivaStyle caused a greater overall color change with no significant difference between the ∆*E* means. There were no differences between T5min/5× and VS10min (*p* = 0.487) or after 24-h storage in saliva between T24h/5× and VS24h (*p* = 0.601).

### Effect of peptide-HAP concentration

The Δ*E* means and standard deviations of gp-3-PH/lc, gp-4-PH/hc, and gp-5-WA after treatment, storage in saliva for 24 h, and restaining for 72 h are listed in Table 4. The greatest Δ*E* mean value after treatment was observed in gp-4-PH/hc (1.81), followed by gp-3-PH/lc (0.51) and the control gp-5-WA, which had no change (0.00). Upon storage in saliva for 24 h, all Δ*E* mean values increased in gp-3-PH/lc (2.05), gp-4-PH/hc (2.06), and gp-5-WA (2.06). Immersion of samples in the staining solution for another 72 h increased the Δ*E* mean values in gp-3-PH/lc (2.65) and gp-4-PH/hc (2.85) and was highest in gp-5-WA (3.07).

**Table 4:** Means and standard deviations of Δ*E* for gp-3-PH/lc, gp-4-PH/hc, and gp-5-WA

Group	Δ*E* after treatment	Δ*E* after storage in saliva for 24 h	Δ*E* after restaining for 72 h
gp-3-PH/lc	0.51 (0.20)a	2.05 (1.00)b	2.65 (0.94)b
gp-4-PH/hc	1.81 (1.57)a	2.06 (1.03)a	2.85 (0.71)a
gp-5-WA	0.00 (0.00)a	2.06 (0.72)b	3.07 (0.93)b

Matching letters within the same row denote no statistically significant difference

Storage in artificial saliva for 24 h significantly affected the color change in gp-3-PH/lc (*p* = 0.004) but was not significant in gp-4-PH/hc (*p* = 0.704). Restaining for 72 h resulted in no difference between the ∆E mean values in gp-3-PH/lc (*p* = 0.142) or gp-4-PH/hc (*p* = 0.145). In contrast, restaining caused a significant difference between the ∆E mean values in gp-5-WA (*p* = 0.041).

### SEM evaluation

**Fig. 2:**
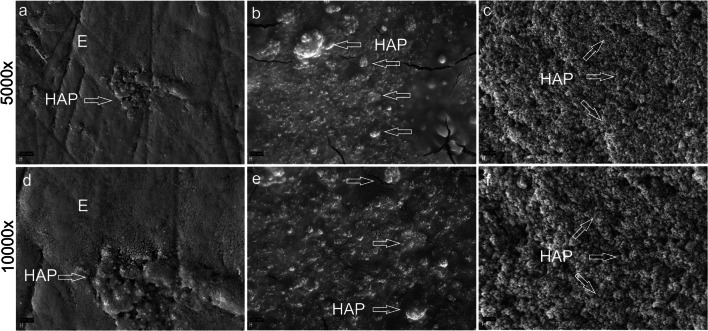
SEM images showing the bovine enamel surface coated with the peptide-HAP suspension of various concentrations at × 5000 (**a–c**) and × 10,000 magnification (**d–f**). In the first group, SEM1-PH/lc, the low concentration peptide-HAP suspension resulted in small quantities of HAP (arrow) on the enamel surface (**E**) resembling islands (**a**, **d**). In the second group, SEM2-PH/hcp, the polymeric form of peptide-HAP, a layer with many HAP particles, and HAP agglomerates (arrows) were detected (**b**, **e**). In the third group, SEM3-PH/hcm, the monomeric form of peptide-HAP, showed a continuous or seamless layer of HAP covering the whole enamel surface (arrows) (**c**, **f**)

In SEM1-PH/lc, HAP was only detected in small quantities, resembling islands of HAP, whereas in SEM2-PH/hcp, the enamel surface was covered with many HAP particles. SEM3-PH/hcm revealed a continuous or seamless layer of HAP covering the whole enamel surface (Fig. 2).

## Discussion

In this study, the null hypothesis can be accepted regarding the application frequency and exposure time in low concentration of peptide-HAP solution compared with a commercial bleaching agent. However, the null hypothesis can be rejected regarding the concentration of the peptide-HAP suspension.

Our findings that low concentration peptide-HAP suspension and the bleaching agent produce similar color changes on bovine teeth indicate that despite their different chemical composition and reactivity with tooth structure, both agents produce similar whitening effects. A slightly stronger color change of the bleaching agent could be due to the longer exposure time of 10 min, as recommended by the manufacturer. We observed that increasing the exposure time of the low concentration peptide-HAP suspension did not influence the tooth whitening effect, which is in favor of the general recommendations of using mouthwashes regularly but only for a short duration of rinsing time [22].

**Fig. 3:**
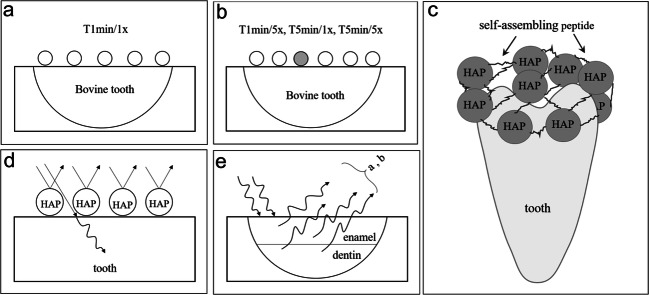
Schematic representation of a physical explanation of the results after more frequent treatment with peptide-HAP suspension (**a**, **b**): less HAP load on the surface represented by the gray HAP particle; thus, the additional whitening effect is poor. Display of the bonding mechanism of P11-4 and HAP to the enamel surface (**c**). Schematic drawing of the light’s behavior on the tooth surface in the presence of HAP particles (**d**). Light’s behavior on the tooth surface. Light is scattered at the HAP underside (**e**)

Common bleaching methods recommend frequent treatment applications to achieve better and more stable results, and most home bleaching methods should be performed once a day for a week [23]. The results of the current study indicated that the whitening effect of the peptide-HAP suspension was not frequency dependent. This can be explained by the assumption that the first exposure of the tooth to the suspension produces an adherent layer of HAP particles that completely cover the enamel surface, preventing further chemical whitening effects upon subsequent exposures (Fig. 3a–c). Therefore, more frequent applications showed only slight color changes. However, more frequent applications of the peptide-HAP suspension may have different results when tested in vivo.

Bleaching agents that contain glycerine have a significant dehydrating effect on bovine teeth [24]. Based on the information given by the manufacturer, Vivastyle Paint On is not a glycerine-based agent but does include ethanol as a solvent. Moreover, Vivastyle Paint On contains carbamide peroxide as an active agent. Some studies have shown that in comparison with carbamide peroxide, pure hydrogen peroxide is released and decomposed more quickly [25].

When comparing the effect of the peptide-HAP suspension with commercial bleaching agents, it is important to consider the duration and frequency of using the peptide-HAP suspension by the patients. Most peroxide-based commercial home bleaching products are used for 1 to 2 h continuously and others overnight. The positive and intensive results obtained after treatment with commercial bleaching agents tend to satisfy patients. We observed that the overall color changes caused by the proposed peptide-HAP suspension did not differ from that caused by the commercial bleaching agent.

Increasing the concentration of the peptide-HAP suspension may significantly promote its whitening effect. Further investigations are needed to determine the optimal concentration of peptide-HAP suspension required to produce an ideal at-home tooth whitening and remineralization suspension. The formed peptide-HAP layer on the surface after treatments with suspensions of varying concentrations may be affected by the pH of the staining solution during restaining. The acidic pH of the solution may dissolve the HAP layer, removing it from the surface and leading to slight darkening of treated samples after restaining. However, it was observed that restaining caused significant color change in only the control group. This might indicate that using the peptide-HAP suspension, regardless of its concentration, may protect against or offset tooth discoloration after restaining.

Curodont Protect includes self-assembling peptides P11-4, which are self-organizing molecules with effective protective characteristics due to combination and stabilization with calcium phosphate. This product is presented as a gel in polymeric form, and the peptides are polymerized to small fibers by the manufacturer. Applying the gel onto the tooth surface, the peptide diffuses into the micro pores of the subsurface and forms a 3D scaffold from the fibers, enhancing the crystallization of HAP [26]. Thus, it acts as a matrix and an attachment medium between HAP particles and enamel. Self-assembling peptide P11-4 was originally utilized for treating initial caries and enhancing remineralization [19]. Our goal was to investigate whether the P11-4 peptide acts as an attachment medium to bond HAP particles to the enamel due to its high affinity for HAP. The anionic groups of the P11-4 peptide side chains attract calcium ions of HAP to the enamel [19, 27].

Calcium phosphate formulations contribute to the precipitation of inorganic crystals onto the enamel surface [28, 29]. Due to this property, we used HAP as a whitening agent, which may boost remineralization and enhance strengthening of enamel during the whitening process [30].

Dental enamel is a highly mineralized tissue that is relatively stable in the normal oral environmental conditions, where saliva plays a role in promoting the balance between dissolution and deposition of minerals [31]. Saliva is crucial for tooth remineralization as it supplies calcium and phosphate ions to build HAP blocks into crystal voids. However, the rate and level of dissolution depends on the concentration of calcium and phosphate ions in saliva and its pH [32]. Moreover, saliva contains a wide range of organic and inorganic compounds that protect and maintain the tooth structure and that may interfere with the whitening process [33].

Some studies have used artificial or natural saliva before treatment to simulate the clinical situation, but there is no universal saliva model to follow [34–36]. Natural saliva acts more effectively to protect the damage caused by bleaching and is a suitable storage medium for in vitro studies [36]. Further storage mediums include purified and mineral water, ethanol, glutaraldehyde, formalin, methanol, chloramine T, salt solution, and mineral oil [37]. Other researchers store their samples in distilled water between bleaching steps, which has the potential to demineralize teeth during the storage period due to ion imbalances [38, 39].

In the current study, we did not store the samples in saliva before treatment to avoid probable interference with the experiment, but after respective treatments in each of the study groups, samples were stored in saliva for 24 h to investigate whether it had any effect on the produced tooth whitening. Our results demonstrated that storage in artificial saliva did not produce any further color changes in the teeth, indicating the chemical stability and reliability of the whitening effect of the peptide-HAP suspension. In all parts of the current study, we used Evian water as a storage medium before treatments and for rinsing the samples. Evian water is not deionized and is a highly standardized water that has a close to natural pH value (7.18). Moreover, Evian water is considered not to be saturated with ions, in contrast to saliva, which could prevent the crystal growth that was not attributed to the treatment.

L^*^ value represents the brightness and is a measure of reflection on HAP particles, while a* and b* values are the measures of attenuation of the inherent color of the tooth under the HAP layer. Increasing the L^*^ value occurs through the reflection of light on the HAP layer and by decreasing light transmission through HAP on the tooth. We assumed that in the presence of the HAP layer on the tooth surface, light is also scattered on the HAP underside. This may lead to reduced light transmission through enamel and dentin and reduction in the a^*^ and b^*^ values measured by the spectrophotometer (Fig. 3d–e).

Measuring the tooth whitening effect can be performed by color assessment using dental shade guides and image analysis of digital dental photographs. Although these clinical methods are more accessible and relatively cost-effective, their accuracy is inadequate, they are sensitive to the measurement environment, and they require a skilled examiner. In contrast, spectrophotometers and colorimeters are considered more reliable, precise, and objective for determining tooth color [40].

Samples were evaluated using a spectrophotometer and a custom made sample holder facilitated to place the samples in a reproducible position in front of the device’s aperture. As a quality control, the test-retest reliability for two repeated measurements was performed. After collecting and analyzing L^*^, a^*^, and b^*^ values; following color measurements; and calculating the corresponding ∆*E* mean values, we observed that all ∆*E* mean values for all conditions were higher than 4, indicating that the overall color changes were perceptible.

The baseline color was measured only after immersion in the staining solution in order to measure whether the different treatments had an effect on the discolored enamel. This method was an attempt to simulate the clinical situation in which a patient would present himself with discolored teeth and seeks a bleaching treatment for improved aesthetics. Thus, our experimental procedures tested whether the application of the peptide-HAP solution would be an acceptable alternative to using a bleaching agent.

Tooth dehydration results in increasing visible and measurable tooth brightness by increasing enamel opacity [41, 42]. Rehydration of dehydrated teeth takes greater than 30 min, but it is possible to return to the baseline color of a tooth after sufficient rehydration [41]. Thus, color measurement procedures should be conducted quickly and before tooth dehydration [43]. During the experiments in the present study, sample dehydration was prevented by continuous storage of the samples in water. We kept the lead time before each measurement as short as possible to avoid unusable results and errors associated with sample dehydration.

SEM images displayed the morphological and structural changes of the enamel surface after treating teeth with peptide-HAP containing suspensions. SEM examination revealed a newly formed surface layer of HAP particles that varied in thickness depending on the peptide-HAP concentration in the different study groups. Increasing the suspension concentration increased the amount of peptide-HAP particles on the tooth surface. The produced layer may improve diffuse light reflection from the surface and is potentially capable of forming a barrier against future acid attacks or discoloration.

In the current study we have not investigated HAP diluted in water because results from previous studies of our research team have found that HAP at higher concentrations such as 44.4 wt% HAP in a nano-hydroxyapatite suspension, and 10–30 wt% tricalcium phosphate have whitening effects [30, 44]. In spite of a smaller HAP concentration (0.5 wt% and 6.25 wt%) in the current study the amount of peptide-HAP adhering to the enamel in the Curodont Protect group (SEM2-PH/hcp) was similar to the higher HAP concentration observed in the previously mentioned studies [30, 44]. Thus, the similar adherence of peptide-HAP to enamel in our study could be attributed to the self-assembling peptide.

The diffuse reflection of light is explained by the peptide-HAP particles adhering to the enamel surface, which may lead to whitening of the teeth. It was previously observed that treatment with HAP-based toothpastes resulted in remineralization of the enamel surface, which made the surface smoother, and the surface layer of HAP increases the diffuse reflection of light, which may result in a considerable increase in brightness [10, 11].

Bovine teeth are the most common alternative for human teeth as there are negligible differences in chemical composition and physical properties between bovine and human enamel. The size of human enamel crystals is slightly smaller than that of bovine enamel crystals, but differences in general are tolerable, and extracted bovine incisors are more accessible than human ones [45–48].

The enamel surface was polished to the remove the aprismatic layer, which contains more minerals than the enamel subsurface because it is less permeable to treatment agents and acidic solutions [49, 50]. The native tooth surface is slightly curved and shines strongly, and due to its shine, the proportion of specular reflection of light is high. The curvature makes the direction in which the light is reflected unpredictable. Although we used an integrating sphere to measure the reflections, we ground the enamel surface flat to avoid this unreliability due to the strong light reflection from the curved surface. Moreover, exogenous discolorations were completely removed by surface grinding.

There are various possible methods for utilizing the proposed suspension. Among the suggested forms is loading in custom made deep drawn splints, which might add cost and time factors, and loading in a toothpaste, which is more convenient and less expensive. Additional studies are needed to determine the most effective concentration of the peptide-HAP suspension for use as an at-home tooth whitening and remineralization agent.

## Conclusions

This investigation draws the following conclusions. Exposure time and application frequency of the peptide-HAP suspension do not affect its tooth whitening potential. The tooth whitening effect of peptide-HAP suspensions is concentration dependent, with greater tooth whitening effects occurring upon using higher concentrations. Applying the peptide-HAP suspension to the tooth surface appears to protect against future tooth discoloration.
